# Structural study of a small molecule receptor bound to dimethyllysine in lysozyme†
†Electronic supplementary information (ESI) available: Fig. S1: crystals of the lysozyme-KMe_2_:sclx_4_ complex grown at different sclx_4_ concentrations. Fig. S2: crystals of the complex grown in the presence of chloride- and sulfate-containing salts. Fig. S3: 1D ^1^H NMR spectra of lysozyme-KMe_2_ in buffer and DMSO mixtures. Table S1: summary of crystallization conditions, data collection and refinement statistics. Movie S1: MD simulation snapshots of sclx_4_ binding to Lys116-Me_2_. See DOI: 10.1039/c4sc02383h



**DOI:** 10.1039/c4sc02383h

**Published:** 2014-10-15

**Authors:** Róise E. McGovern, Brendan D. Snarr, Joseph A. Lyons, James McFarlane, Amanda L. Whiting, Irina Paci, Fraser Hof, Peter B. Crowley

**Affiliations:** a School of Chemistry , National University of Ireland Galway , University Road , Galway , Ireland . Email: peter.crowley@nuigalway.ie ; Tel: +353 91 49 24 80; b Department of Chemistry , University of Victoria , British Columbia V8W 3V6 , Canada; c School of Biochemistry and Immunology , Trinity College Dublin , Dublin , Ireland

## Abstract

Lysine is a ubiquitous residue on protein surfaces. Post translational modifications of lysine, including methylation to the mono-, di- or trimethylated amine result in chemical and structural alterations that have major consequences for protein interactions and signalling pathways. Small molecules that bind to methylated lysines are potential tools to modify such pathways. To make progress in this direction, detailed structural data of ligands in complex with methylated lysine is required. Here, we report a crystal structure of *p*-sulfonatocalix[4]arene (sclx_4_) bound to methylated lysozyme in which the lysine residues were chemically modified from Lys-NH_3_^+^ to Lys-NH(Me_2_)^+^. Of the six possible dimethyllysine sites, sclx_4_ selected Lys116-Me_2_ and the dimethylamino substituent was deeply buried in the calixarene cavity. This complex confirms the tendency for Lys-Me_2_ residues to form cation–π interactions, which have been shown to be important in protein recognition of histone tails bearing methylated lysines. Supporting data from NMR spectroscopy and MD simulations confirm the selectivity for Lys116-Me_2_ in solution. The structure presented here may serve as a stepping stone to the development of new biochemical reagents that target methylated lysines.

## Abstract 2

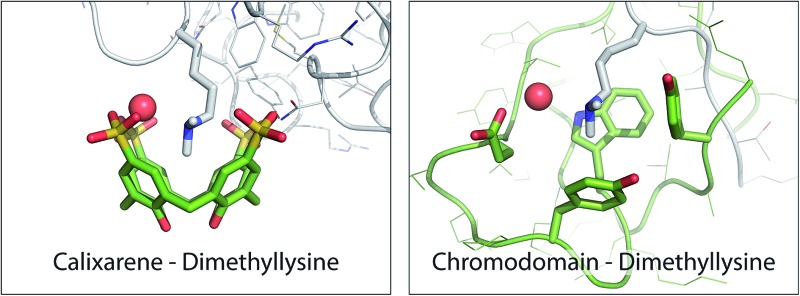
X-ray crystallography reveals how a calixarene can bind to dimethyllysine to form a complex with features similar to the aromatic cage motif of a chromodomain bound to a histone tail.

## Introduction

Lysine is one of the most abundant residues on protein surfaces. With four methylenes and an epsilon amino group it is a cation of substantial conformational flexibility. Although native lysine is generally excluded from protein–protein interfaces^1^ numerous post-translational modifications^2,3^ produce a variety of functional groups with altered interaction properties. In particular, lysine methylation to the mono-, di- or trimethylated amine yields hotspots for protein interactions. Prominent examples include the methyllysines of histone tails that insert into aromatic cage motifs of chromatin remodelling enzymes.^2,4–6^ In recent years many other (non-histone) proteins have been shown to contain methylated lysines,^7,8^ though the roles of these modifications remain largely uncharacterized.

There is growing interest in the development of small molecule receptors that bind to lysine^9–19^ and its methylated derivatives and analogues.^20–30^ Synthetic ligands for methylated lysines have potential applications as inhibitors of protein–protein interactions and can be used as reagents in biochemical assays^26,31^ and cell biology.^32^ In certain cases it has been shown that lysine receptors bind to the methylated side chain with an affinity that is greater by several orders of magnitude and equilibrium dissociation constants (*K*_d_) of ∼10 μM have been reported for peptides containing trimethyllysine.^15,24,29^ The anionic *p*-sulfonatocalix[4]arene^33^ (sclx_4_) and its analogues^34^ have proven to be particularly useful for protein surface recognition^17,35^ and/or complexation with lysine and methylated lysine.^10,15^ A recent study of sclx_4_ interactions with cytochrome *c* provided some of the first structural evidence of lysine recognition by a small molecule receptor.^17^ And the structure of a phosphate-tweezers bound to a lysine in the 14-3-3 protein^18^ further corroborated the use of supramolecular receptors for protein surface recognition.^36,37^ Despite the growing literature on ligand binding to methylated lysines,^27–32^ the structural characterization of a synthetic receptor bound to methylated lysine in a protein is completely lacking.

To gain structural knowledge of the interaction between a small molecule ligand and a protein bearing post-translationally modified lysines we solved the crystal structure of sclx_4_ in complex with dimethylated lysozyme (lysozyme-KMe_2_). The complex was further characterized by NMR spectroscopy and molecular dynamics simulations. We identified a surprisingly selective binding of the calixarene at one of six possible dimethyllysine residues. This selectivity was rationalized in terms of the local chemical environments of the dimethyllysines. A second binding site at Arg14 was also found in the crystal structure.

## Results and discussion

### Choice of model system

**Fig. 1:**
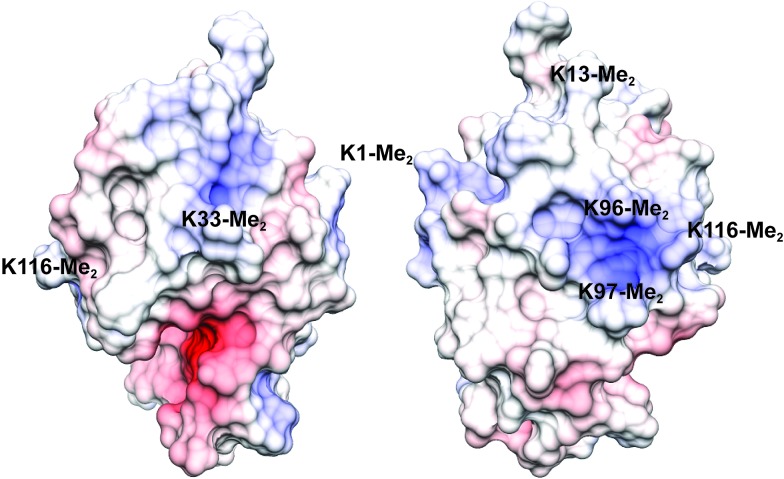
The electrostatic potential surface (adaptive Poisson–Boltzmann solver) of lysozyme-KMe_2_ with positive and negative patches coloured blue and red, respectively (the two views are related by a 180° rotation). Labels indicate the approximate locations of each of the six dimethyllysine residues.

Lysozyme is a highly-characterized model protein that is frequently used for ligand binding studies.^38–41^ Moreover it is a workhorse for structural studies of lysine methylation^42–46^ with well-established protocols for dimethylation by reductive alkylation which modifies lysines and the N-terminus. With a high proportion of lysine/arginine side chains and an overall positive electrostatic potential (pI ∼10, Fig. 1) lysozyme is suited to binding the anionic sclx_4_.^35^

### Calixarene binding in solution

The presence of sclx_4_ resulted in the immediate precipitation of lysozyme-KMe_2_. Precipitation occurred at μM – mM protein concentrations and crystals grew at protein and ligand concentrations as low as 20 and 1 μM, respectively (Fig. S1†) and in the presence of different sulfate- and chloride-containing salts (Fig. S2†). Notably, crystal growth occurred in the absence of precipitants such as PEG or ammonium sulfate. These data suggested a relatively high affinity interaction (*K*_d_ ∼μM).

**Fig. 2:**
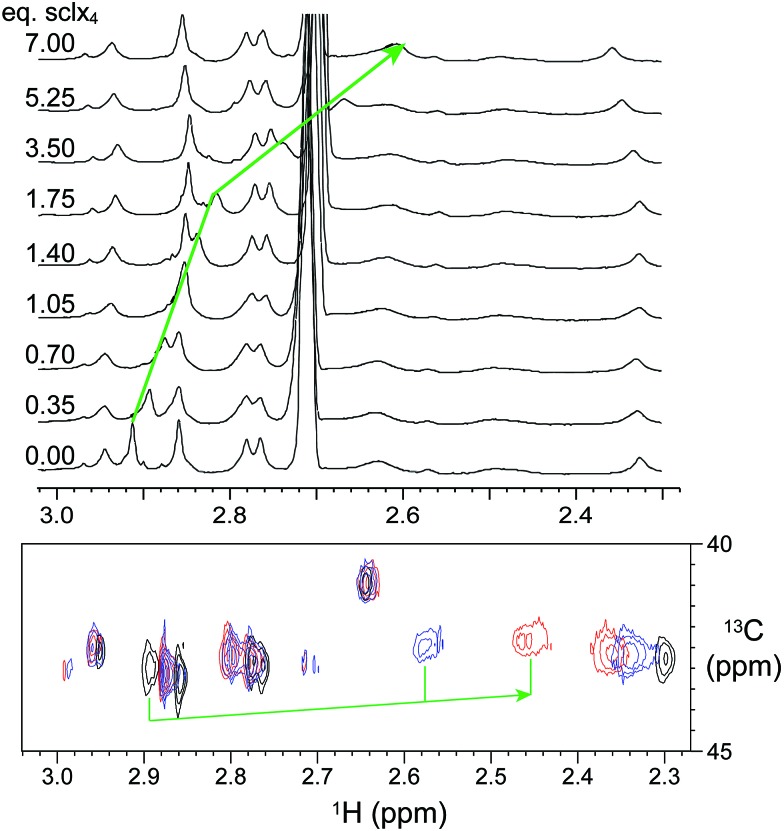
NMR spectroscopic characterization of sclx_4_ interactions with lysozyme-KMe_2_. Upper panel, 1D ^1^H NMR spectra (showing the region corresponding to –NMe_2_ resonances) of dimethylated lysozyme in the presence of 0–7 equivalents of sclx_4_. Lower panel, ^1^H-^13^C HSQC spectra of ^13^C-labeled dimethylated lysozyme (black contours) in the presence of 7 (blue) and 10 (red) equivalents of sclx_4_. The green arrows indicate the upfield shifts of the resonance assigned to Lys116-Me_2_. A smaller downfield shift was observed for the Lys1-Me_2_ resonance. The signal at ∼2.7 ppm corresponds to DMSO. Samples were in 40 mM sodium phosphate, 10% D_2_O, and 20% DMSO-*d*_6_, pH 7.4.

Attempts to characterize the complex in water/buffer were thwarted by precipitation. Thus, NMR spectroscopy was performed on protein samples in water–DMSO mixtures. Apart from small changes, the ^1^H NMR spectrum of lysozyme was largely unaffected by 20% DMSO (Fig. S3†) indicating that the protein was stably-folded under these conditions^47^ (>50% DMSO is required to unfold lysozyme).^48^ Titrations were performed by the addition of μL volumes of a stock solution of sclx_4_ and complex formation was monitored by collecting 1D ^1^H and 2D ^1^H-^13^C HSQC spectra (Fig. 2). A single resonance at ∼2.92 ppm, assigned to the N^ε^Me protons of Lys116-Me_2_,^46^ demonstrated a large upfield chemical shift. The nature of this chemical shift perturbation was consistent with ring current effects induced by the phenyl rings of the calixarene cavity and suggests that the dimethylamino group was buried inside sclx_4_.^15^ A plot of the chemical shift changes as a function of the ligand concentration resulted in a shallow curve (data not shown) that was unsuited to an accurate *K*_d_ determination. It was not possible to reach saturation as sample precipitation occurred at >10 equivalents of ligand. Similar chemical shift perturbations were observed in 10% DMSO, although precipitation occurred at lower sclx_4_ concentrations. This indicates that DMSO serves to reduce precipitation without impacting the binding selectivity.

A small downfield shift for the resonance assigned to Lys1-Me_2_ (∼2.32 ppm)^46^ was observed at >3 equivalents of sclx_4_ suggesting that a weaker interaction occurred at this site. However, the downfield shift indicated that encapsulation of the dimethylamino did not occur in this case. It is reasonable to assume that the probability of weak interactions at the N-terminal Lys1-Me_2_ is greater than at the other dimethyllysines due to the relatively higher accessibility of this residue and the presence of two dimethylamines (at the N^ε^ and the N^α^ atoms).

### Crystal structure of the lysozyme-KMe_2_:sclx_4_ complex

**Fig. 3:**
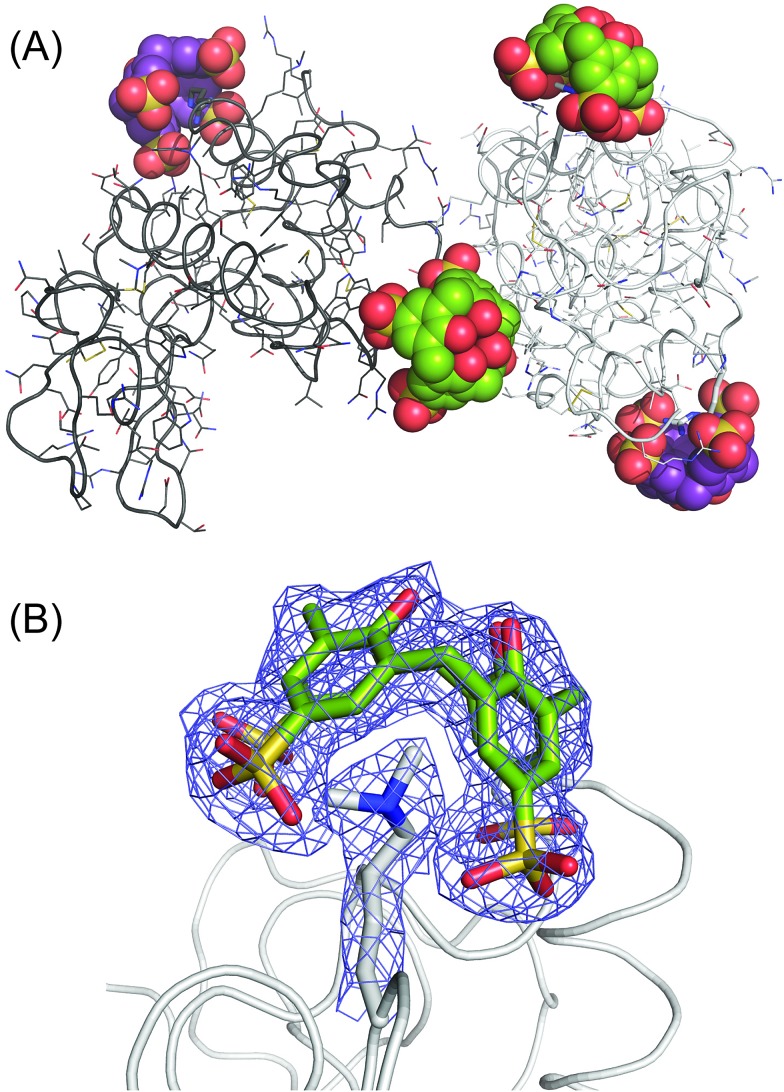
The lysozyme-KMe_2_:sclx_4_ complex. (A) The asymmetric unit comprises two molecules of lysozyme-KMe_2_ (rendered as light and dark grey ribbons) and four molecules of sclx_4_. The dimethyllysine-binding and the arginine-binding calixarenes are coloured green and purple, respectively. (B) The calixarene binding site at Lys116-Me_2_ showing the 2*F*_o_ – *F*_c_ electron density map around the Lys116-Me_2_ side chain and sclx_4_ (contoured at 1.0*σ*). See Fig. 4A for a detailed view of the sclx_4_–Lys116-Me_2_ interaction. The crystals used for this structure were grown from a 1 : 5 protein–ligand mixture (Table S1†).

Crystals of the sclx_4_ complex with lysozyme-KMe_2_ grew under similar conditions and in the same space group as native lysozyme^35^ but with a ∼50% smaller unit cell (Table S1†). Two almost identical structures (1.9 and 2.2 Å) were refined. The asymmetric units comprised two molecules of lysozyme-KMe_2_ and four molecules of sclx_4_ (Fig. 3A). Similar protein–ligand interactions were observed in each lysozyme-KMe_2_ molecule. In agreement with the NMR data (Fig. 2) the side chain of Lys116-Me_2_ was encapsulated by sclx_4_. A second ligand was observed to bind Arg14. As noted in previous structures, the calixarene appeared to act like “molecular glue” at protein–protein interfaces in the crystal.^17,35^

**Fig. 4:**
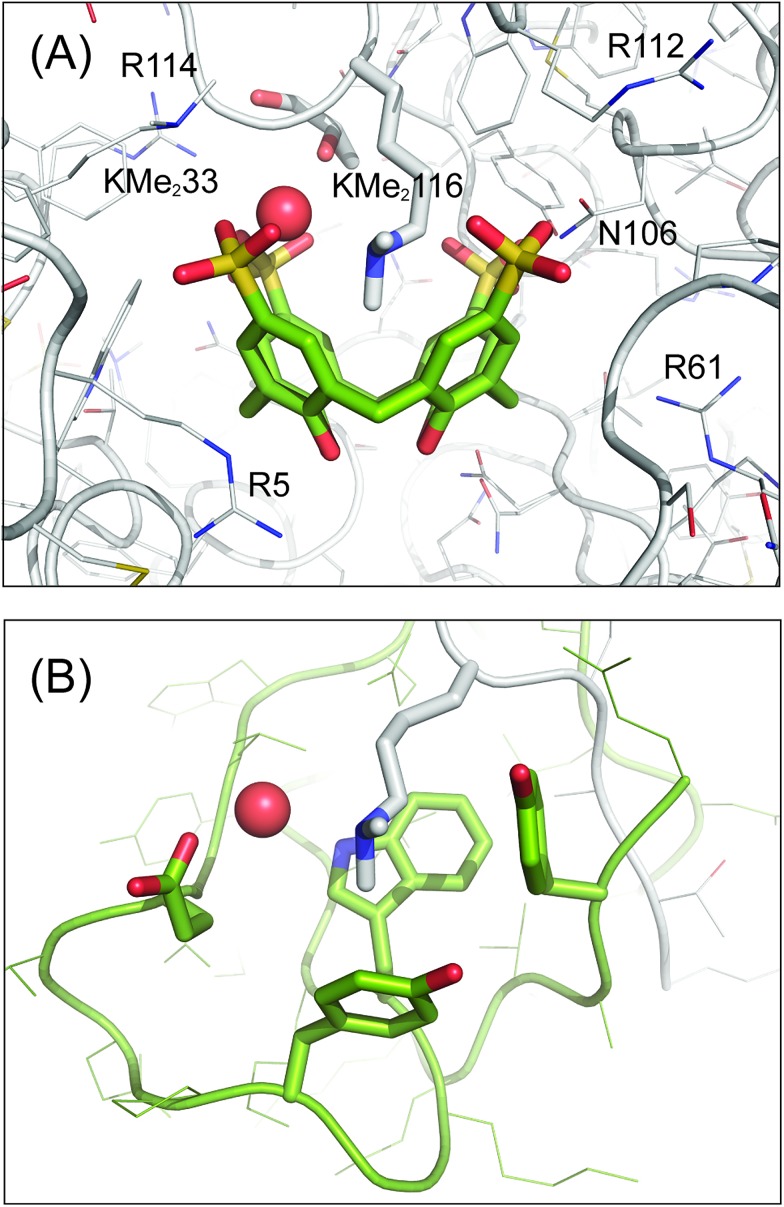
Sclx_4_ mimics the aromatic cage motif for binding dimethyllysine. (A) Detailed view of the sclx_4_ complex with Lys116-Me_2_. Neighbouring cationic side chains and Asn106 are labelled, see main text for details. (B) The aromatic cage (green) in the chromodomain of HP1 bound to dimethylysine in a histone H3 tail peptide (PDB ; 1KNA).^5^ The chromodomain side chains Tyr24, Trp45, Tyr48 and Glu52 are shown as sticks. In both structures the dimethyllysine amino is solvated by a water molecule (red spheres), which is hydrogen bonded to one or more acidic substituents on the receptor. The proteins are shown as C^α^ traces with side chains as lines or sticks and the C atoms of sclx_4_ are green.

The sclx_4_–dimethyllysine interaction involves one of the methyl groups of Lys116-Me_2_ inserted into the core of the calixarene, which behaves like a four-walled aromatic cage (Fig. 4A). The distance between the methyl carbon (C^η^) and the centroids of the calixarene phenyl rings (3.5–3.9 Å) is consistent with cation–π interactions.^6,24,49^ Short range contacts occur between the second methyl group of Lys116-Me_2_ and two of the phenyl rings. Interestingly, this methyl is also in van der Waals contact (3.5 and 3.8 Å) with oxygen atoms of two sulfonates, hinting at the possibility of CH···O salt bridges. Water too plays a role in the coordination environment of the dimethyllysine. The tertiary amino nitrogen is 2.7 Å from a water molecule, which is neatly positioned between two sulfonates, indicative of water-mediated salt bridge interactions. The energetic contributions to sclx_4_–dimethyllysine binding are expected to be dominated by cation–π interactions (rather than salt bridges)^6^ and a contribution from the hydrophobic effect is also to be expected considering the water-bearing capacity of the calixarene cavity.^17,33^ These features of the sclx_4_–dimethyllysine interaction are further interesting in terms of their resemblance to how proteins such as chromodomains bind to methylated lysines in histone tails. An aromatic cage, typically comprising three aromatic side chains, provides a pocket for the methylated lysine side chain, which remains partially solvated (Fig. 4B).^2,5^

Remarkably the conformation of Lys116-Me_2_ bound to sclx_4_ was almost identical to the side chain conformation observed in the original structure of dimethylated lysozyme (PDB ; 132L)^43^ even though the crystals were grown from completely different conditions [PEG and low salt at pH 6.0 (Table S1†) *versus* 1.5–2.2 M MgSO_4_ at pH 8.0]. The only difference was a rotation about the C^δ^–C^ε^ bond, which increases the accessibility of the dimethylamino group in the sclx_4_-bound structure.

### Lysine *versus* dimethyllysine binding and selectivity

Substantial differences were observed for the binding of sclx_4_ to lysine and to dimethyllysine. In the case of cytochrome *c*, the lysine side chains were fully encapsulated by the calixarene cavity such that all four methylene groups were in van der Waals contact with one or more of the calixarene phenyl rings.^17^ To accommodate the entire side chain in this fashion the calixarene adopted a pinched cone conformation and the lysine amino group was positioned off-centre bringing it close to two of the sulfonates. In the case of dimethyllysine, one of the methyl groups is positioned in the centre of the calixarene such that the amino nitrogen is equidistant from all four sulfonates. Only the C^η^ and C^ε^ atoms make van der Waals contact with the calixarene phenyls while the remainder of the side chain protrudes from the cavity (Fig. 4A). Thus it appears that this complex favours a regular cone conformation of sclx_4_, which maximises cation–π bonds with the dimethylamino group.^6,49^ Similar interactions were found previously in complexes of sclx_4_ with tetramethylammonium cations.^22^

**Fig. 5:**
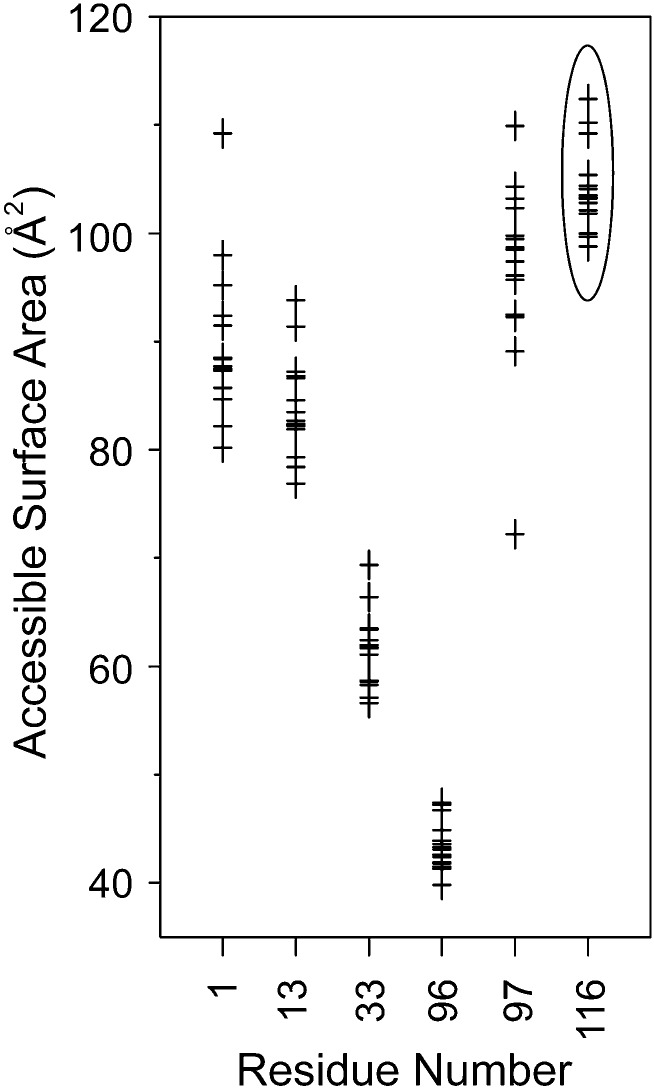
The accessible surface area of the lysine residues in 15 high resolution crystal structures of lysozyme.^35^ Lys116 (highlighted by an ellipse) was on average the most accessible residue. The dimethylated form of Lys116 was selectively bound by sclx_4_ (Fig. 2 and 4A).

These observations help to explain the selectivity for Lys116-Me_2_, which projects out from the protein surface and has Asn106, Arg112 and Gly117 as neighbours. A hydrogen bond (Lys116 to Asn106) in the native protein is absent in the dimethylated protein where the Lys116-Me_2_ side chain flips into the calixarene cavity and Asn106 hydrogen bonds to two of the sulfonates (Fig. 4A). The steric accessibility of most of the other Lys-Me_2_ side chains is significantly lower, which may preclude sclx_4_ binding. Lys1-Me_2_ forms a cation–π interaction with Phe3, and salt bridges with Glu7 and the sclx_4_ bound to Arg14; Lys13-Me_2_ is screened by the carboxylates of Asp18 and *C*-terminal Leu129; Lys33-Me_2_ is flanked by Asn37 and the bulky aromatics Phe34, Phe38 and Trp123, only the amine is accessible and it forms a salt bridge with a sclx_4_ sulfonate; Lys96-Me_2_ is buried and forms weak cation–π interactions with both Tyr20 and the sclx_4_ bound to Arg14; while Lys97-Me_2_ forms a salt bridge with Asp101. Similar conformations of the lysine side chains are present in the native lysozyme structure. To substantiate these observations the solvent accessible surface area (ASA) was calculated for each lysine in a dataset of 15 high resolution structures of lysozyme.^35^ On average, Lys116 was the most accessible lysine (Fig. 5). While Lys97 has a similar ASA, it may be the differences in the local charge that tips the scales in favour of sclx_4_ binding to Lys116. Considering charged groups within an 8 Å radius, Lys97-Me_2_ forms a salt bridge with Asp101 while Lys116-Me_2_ is neighboured by Arg112. The higher positive charge of the latter region will afford a greater attraction for the anionic sclx_4_. To investigate this hypothesis we performed MD simulations of sclx_4_ binding to Lys-Me_2_ side chains.

### Molecular dynamics of sclx_4_ binding to Lys-Me_2_

**Fig. 6:**
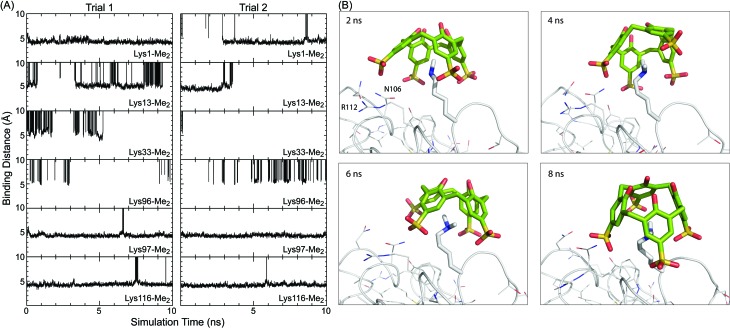
(A) Binding distance (see main text for description) between sclx_4_ and individual Lys-Me_2_ side chains over the course of two MD simulations of 10 ns duration. Time points when the ligand was unbound are off the scale (0–10 Å). (B) Representative snapshots of ligand binding at Lys116-Me_2_. The protein is represented as the C^α^ trace with side chains and Lys116-Me_2_ shown as lines and sticks, respectively. Refer to the movie (ESI†) for a more comprehensive view of the binding conformations.

Duplicate simulations (10 ns duration) were performed on sclx_4_ binding to each of the six Lys-Me_2_ side chains. The goal was to identify the structural features that distinguish Lys116-Me_2_ from the other five potential binding sites. Two main features were considered; (1) the accessibility of the side chain, and (2) the local interactions, including those between the ligand substituents and peripheral residues. Site-specific information was determined from the molecular dynamics trajectories, which were examined primarily in terms of the “binding distance” as a means to quantify the degree of encapsulation of the Lys-Me_2_ in the sclx_4_ cavity. This was defined as the distance from the Lys-Me_2_ N^ε^ atom to the best-fit plane through the methylene bridge carbons of the calixarene. Fig. 6A shows the evolution of the binding distance at the six sites, during two simulation trials. The trends explicitly illustrate the large differences in the potential for complex formation at the different sites. Lys13-Me_2_, Lys33-Me_2_ and Lys96-Me_2_ did not form temporally stable complexes with sclx_4_. In contrast, Lys97-Me_2_ and Lys116-Me_2_ were stably bound for almost the entire trajectory of each simulation consistent with the greater accessibility of these residues (Fig. 5). At a binding distance of 4–5 Å the dimethylamino is positioned deep within the calixarene cavity where it forms cation–π interactions. The complex at Lys1-Me_2_ was similar though it was unbound over ∼3 ns of trial 2, suggesting a lower stability interaction. Attempts to discriminate the sites at Lys1-Me_2_, Lys97-Me_2_ and Lys116-Me_2_ using calculated binding energies were unsuccessful, likely due to the well-documented incompatibility of the MM-PBSA end-state free energy method with highly charged systems.^50,51^

Further analysis was focused on the complexes at Lys97-Me_2_ and Lys116-Me_2_. Representative snapshots of the complexes at Lys116-Me_2_ (Fig. 6B, see also the MD movie, ESI†) show the potential role for both Arg112, which can form salt bridge interactions with one of the calixarene sulfonates, and Asn105, which can hydrogen bond to the sulfonates. The two residues interact with the calixarene outer rim alternatively, with the Arg112 in binding position 25–40% of the time. These observations were corroborated by the crystal structure. In protein chain B there is a salt bridge between Arg112 and one of the calixarene sulfonates, while in chain A an alternative rotamer is present and Arg112 forms an intramolecular hydrogen bond with the protein backbone. The amide of Asn106 is positioned equidistantly from two sulfonates in the site at chain A, while in chain B only the amide N can form a hydrogen bond with sclx_4_. Similarly, calixarene binding at Lys97-Me_2_ can involve salt bridge interactions with Arg21 (80–90% of the simulation time) and hydrogen bonds with Asn93 with a lower incidence. However, as noted above Lys97-Me_2_ can also salt bridge with Asp101. The MD data suggests that, once complexed, Lys116-Me_2_ and Lys97-Me_2_ can interact with sclx_4_ in a similar fashion. Selectivity of the calixarene to Lys116-Me_2_ is due to the greater steric accessibility at that site. This is supported by the relative values of ASA averages collected during the solution-phase simulations (data not shown), which were in agreement with the crystallographic data (Fig. 5), although ASA values in solution tend to be larger than in solid state due to the enhanced conformational freedom.

### Arginine binding by sclx_4_

While the selectivity of sclx_4_ for Lys116-Me_2_ can be rationalised in terms of steric accessibility, there remains the question of why binding also occurred at Arg14 in the crystal structure (Fig. 3). This question is interesting for two reasons. (1) NMR-derived binding curves for the interaction of sclx_4_ with free amino acids have revealed a ∼50-fold greater affinity for Lys-Me_2_ over Arg.^15^ Thus, it might be expected that sclx_4_ would bind only Lys-Me_2_ side chains in lysozyme-KMe_2_. However, complex formation at Arg14 (instead of, for example, at Lys13-Me_2_) reinforces the fact that protein surfaces, with their complex topologies and chemistries, can greatly alter the affinity of ligand binding. (2) In a crystal structure of native lysozyme and sclx_4_ the ligand was bound at Arg128, the most sterically accessible arginine residue.^35^ In lysozyme-KMe_2_, Arg14, the second most accessible Arg residue was selected by sclx_4_. Arg128 provides additional longer range (∼8 Å) interactions to the sulfonates. It can be concluded that the affinities of sclx_4_ for Arg14 and Arg128 are closely matched and the particular complex that prevails in the crystal structure depends on the crystal packing environment where the calixarene mediates protein–protein contacts.^17,35^

## Conclusions

The data presented here illustrate how a protein containing dimethyllysine can be non-covalently modified by a small molecule receptor. Using a combination of X-ray crystallography, NMR spectroscopy and MD simulations we have shown how the symmetric and anionic sclx_4_ selectively binds to a single dimethylated lysine on the surface of a globular, folded protein. We note structural and chemical similarities between the complexes of dimethyllysine bound to the *simple* calixarene or to the aromatic cage motif of a chromodomain. This data will likely benefit the design of synthetic receptors for proteins (including histones) that contain methylated lysines.

## Experimental

### Materials

Hen egg white lysozyme (62971 Fluka) was dimethylated by using dimethylamine borane complex and formaldehyde according to published methods.^43–46^ Electrospray ionization mass spectrometry data (Waters LCT Premier XE) for lysozyme (14,302.2 Da) and dimethylated lysozyme (14,498.0 Da) indicated complete dimethylation of all six lysines and the N-terminus. ^13^C-formaldehyde was used to prepare dimethylated samples for ^13^C NMR spectroscopy. Chemically-modified protein was purified by carboxymethyl (GE Healthcare) ion exchange chromatography prior to the crystallization experiments.

### NMR spectroscopy

1D ^1^H and 2D ^1^H-^13^C HSQC spectra were acquired on a Bruker AV500 operating at 500 MHz and 25 °C. Protein samples of 0.3–10 mM lysozyme-KMe_2_ in 40 mM sodium phosphate, 10% D_2_O, and 20% DMSO-*d*_6_ at pD = 7.0 (pH 7.4) were titrated with μL volumes of a 0.65 M stock of sclx_4_ in the same solution.

### Crystallization and X-ray structure determination

The hanging drop vapour diffusion method was used for crystallization at 20 °C. Co-crystals of lysozyme-KMe_2_ and sclx_4_ were grown from similar conditions to those reported for lysozyme.^35^ The drops were prepared by combining 1 μL volumes of protein, sclx_4_ and the reservoir solution (Table S1†). Diffraction data for the lysozyme-KMe_2_:sclx_4_ single crystals were collected at the ESRF (BM14, MarCCD detector, *φ* scans of 1° over 180° to a resolution of 1.9 Å) and at the Swiss Light Source (X10SA, 10 μm minibeam, Pilatus 6M detector, *φ* scans of 0.5° over 180°, to a resolution of 2.2 Å). Data processing and scaling were performed in MOSFLM^52^ and SCALA,^53^ respectively or in xia2 (ref. 54) using XDS,^55^ XSCALE and SCALA. See Table S1† for the data collection and refinement statistics. The structures were solved by molecular replacement in PHASER. Refinement and manual rebuilding were performed in REFMAC5 as implemented in CCP4 (ref. 56) and COOT,^57^ respectively. Solvent molecules were placed automatically using ARP/wARP^58^ and refinement was continued until no features remained in the *F*_o_ – *F*_c_ difference maps. Molprobity^59^ was used to check the structure quality. Coordinates and structure factors were deposited in the Protein Data Bank with the accession codes ; 4PRU (2.2 Å) and ; 4N0J (1.9 Å). The protein–ligand and protein–protein interfaces were analysed in COOT.

### Molecular dynamics of protein–calixarene interactions

Binding dynamics were followed using classical molecular dynamics over 10 ns intervals, at a temperature of 300 K. To reduce the introduction of bias a structure of native lysozyme (PDB 3RZ4) was used for the initial coordinates. This structure was modified with newly parameterized Lys-Me_2_ residues replacing all six of the lysines. Dimethyllysine was not available in the standard AMBER residue library so the parameters were retrieved from the literature.^60^ Partial charges were derived from gas phase optimized HF/6-31*G calculations in Gaussian09, and fit in the preparatory program Antechamber using the RESP charge fitting method. The remaining parameters for the nonstandard amino acids were obtained from Antechamber and fit to the AMBER ff10 force field. Parameters for the calixarene were fit to the general AMBER force field for small organic molecules. The ligand structure was minimized in explicit TIP3P water prior to being placed with the protein for simulation. The calculations used explicit TIP3P water and Cl^–^ counter-ions added to charge neutrality.^61,62^ Duplicate protein–calixarene complexes were generated for each candidate Lys-Me_2_ site by combining the equilibrated protein and the minimized sclx_4_ structure. The ligand was placed approximately 7–10 Å above the Lys-Me_2_ side chain and the complex was allowed to equilibrate. The binding energy at each site was calculated using the MM-PBSA method (implicit solvent).^50,51^ Docking was also monitored through calculations of the average binding distance between the dimethylamino group and the plane of the calixarene methylene bridge carbons.
