# Supplementary material for: Structural study of a small molecule receptor bound to dimethyllysine in lysozyme
Source: Chem Sci. 2014 Oct 15;6(1):442–9. doi: 10.1039/c4sc02383h (PMC4266562; doi:10.1039/c4sc02383h)
Supplement: Supplementary file 1 [file SC-006-C4SC02383H-s001.pdf]

Electronic Supplementary Material (ESI) for Chemical Science.
This journal is © The Royal Society of Chemistry 2014

SUPPORTING INFORMATION

Structural study of a small molecule receptor bound to dimethyllysine in lysozyme

Róise E. McGovern,[a] Brendan D. Snarr,[b] Joseph A. Lyons,[c] James McFarlane,[b] Amanda L. Whiting,[b] Irina Paci,[b] Fraser Hof[b] and Peter B. Crowley*,[a]

[a]School of Chemistry, National University of Ireland Galway, University Road, Galway, Ireland.

[b]Department of Chemistry, University of Victoria, British Columbia, V8W 3V6, Canada.

[c]School of Biochemistry and Immunology, Trinity College Dublin, Dublin, Ireland.

*Correspondence to: peter.crowley@nuigalway.ie, +353 91 49 24 80

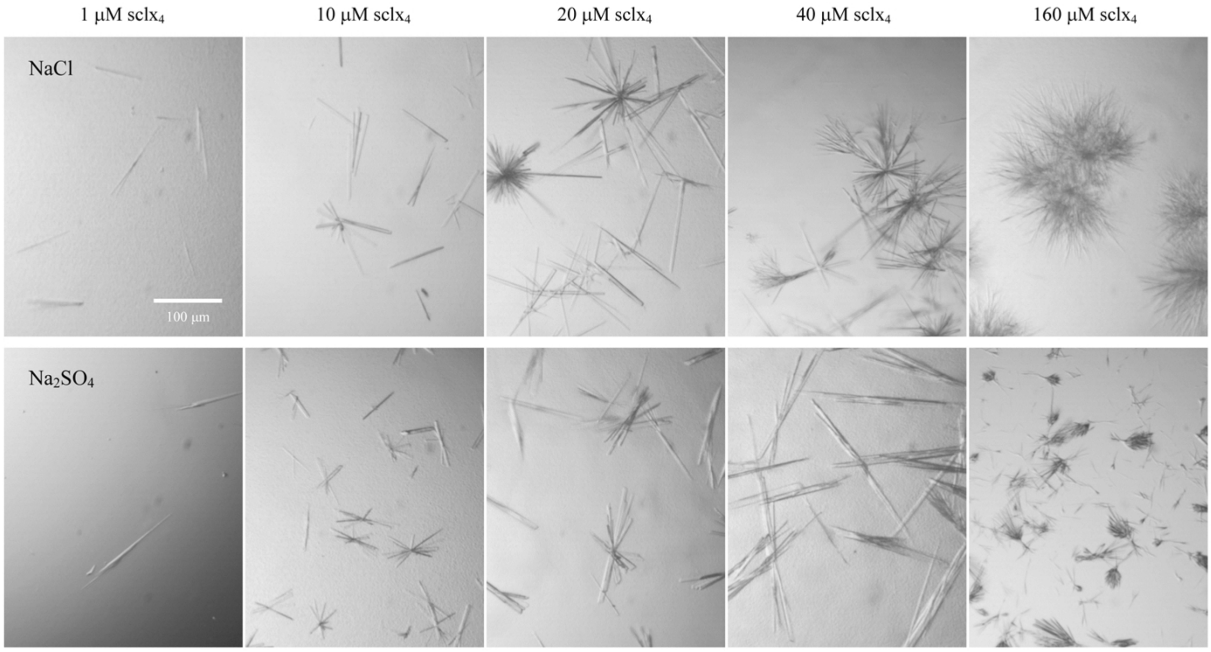

Figure S1. Crystals of the lysozyme-KMe$_2$:sclx$_4$ complex grew in the presence of different concentrations of sclx$_4$. The crystallization drops contained 20 μM lysozyme-KMe$_2$, 1-160 μM sclx$_4$, 20 mM NaCl or Na_2SO_4 and 10 mM $(CH_3)_2AsO_2Na$ (cacodylate) at pH 6. There was no precipitant (*e.g.* PEG or ammonium sulfate) in the drops. Crystals were evident two days after the experiment set up.

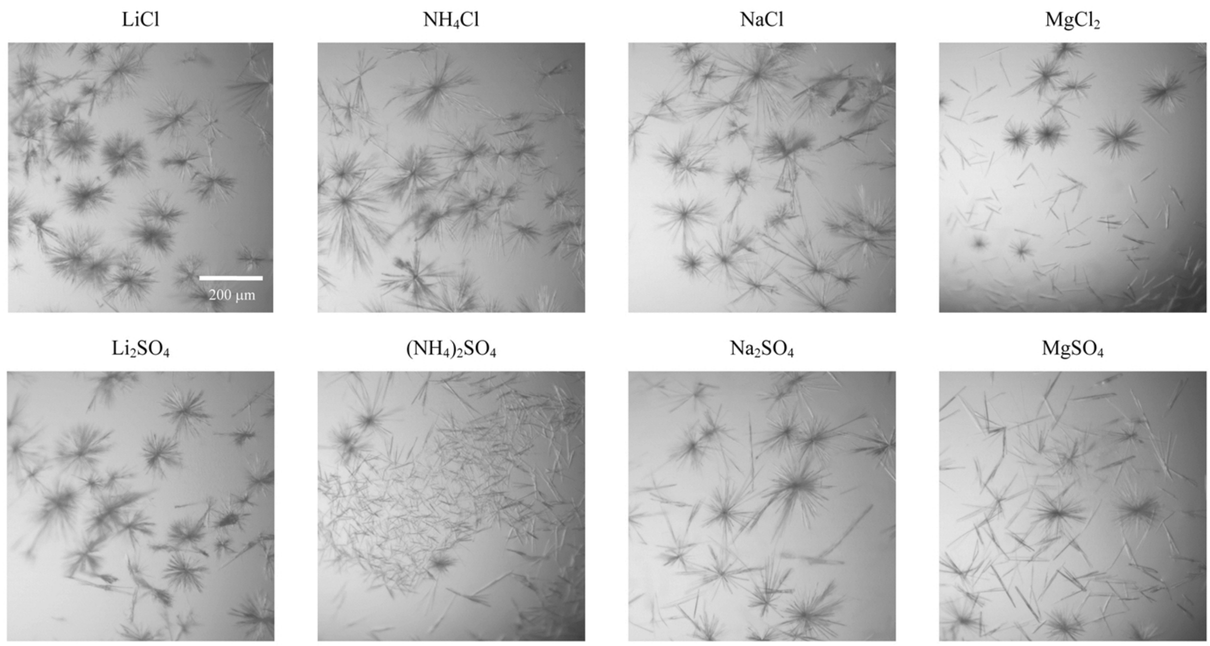

Figure S2. Crystals of the lysozyme-KMe_2:$sclx_4$ complex grew in the presence of different chloride- and sulfate-containing salts. The crystallization drops contained 20 µM lysozyme-KMe_2, 10 µM $sclx_4$, 20 mM salt and 10 mM $(CH_3)_2AsO_2Na$ (cacodylate) at pH 6. There was no precipitant (*e.g.* PEG or ammonium sulfate) in the drops. Crystals were evident two days after the experiment set up.

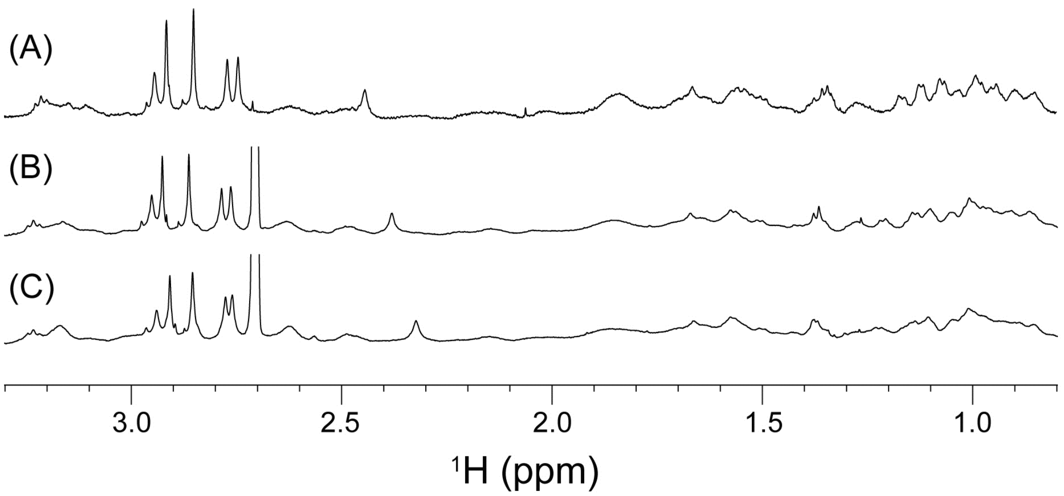

Figure S3. 1D ^{1}H NMR spectra (showing the region corresponding to -NMe_2 resonances and further upfield) of lysozyme-KMe_2 in (A) 40 mM sodium phosphate, pH 7.4, (B) 40 mM sodium phosphate, 10 % DMSO-d_6, pH 7.4, and (C) 40 mM sodium phosphate, 20 % DMSO-d_6, pH 7.4. The signal at ~2.7 ppm corresponds to DMSO.

Table S1. Summary of crystallization conditions, data collection and refinement statistics

	Crystallization Conditions[a]	
[protein], [sclx$_4$] (mM)	0.5, 2.5	0.5, 4.3
PEG 8000 (%)	6	18
Buffer, $(CH_3)_2AsO_2Na$	0.1 M, pH 6.1	0.05 M, pH 6.3
Salts	0.05 M NaCl, 0.1 M $MgCl_2$	
	Data Collection[b]	
Space group	$P2_1$	$P2_1$
Cell constants	a = 45.85 Å<br>b = 30.20 Å<br>c = 94.21 Å<br>$\alpha = \gamma = 90°$<br>$\beta = 96.31°$	a = 44.04 Å<br>b = 30.28 Å<br>c = 95.01 Å<br>$\alpha = \gamma = 90°$<br>$\beta = 96.62°$
Resolution (Å)	31.32-1.90 (1.95-1.90)	39.41-2.20 (2.26-2.20)
Wavelength (Å)	0.95372	1.033190
Unique reflections	20709 (19591)	44504 (13557)
Multiplicity	3.6 (3.5)	3.3 (3.3)
I/σ	9.2 (2.6)	7.9 (3.7)
Completeness (%)	94.6 (90.1)	99.6 (98.9)
R_{merge}[c] (%)	8.4 (41.1)	13.0 (73.4)
Solvent content (%)	45.02	49.53
	Refinement	
PDB ID	4N0J	4PRU
R_{factor} (%)	16.77	18.00
R_{free} (%)	21.04	23.52
rmsd[d] bonds (Å)	0.01	0.01
rmsd angles (°)	1.19	1.53
# molecules in asymmetric unit		
Protein	2	2
Sclx$_4$	4	4
Na^+	1	-
Mg^{2+}	2	-
Cl^-	3	-
Glycerol	5	2
Solvent	191	97
Average B factors[e] (Å^2)		
Protein	13.12	27.57
Sclx$_4$	17.79	33.56
Na^+	16.85	-
Mg^{2+}	19.13	-
Cl^-	18.12	-
Glycerol	25.64	38.37
Solvent	23.75	30.65
Ramachandran analysis[f]		
% residues (favoured regions)	97.95	98.0
% residues (allowed regions)	100.0	98.5

[a]The crystallization drops comprised 1 μL each of the protein, ligand and reservoir solution; [b]Values in parentheses correspond to the highest resolution shell; [c]$R_{merge} = \sum_{hkl} \sum_i |I_i(hkl) - \langle I(hkl)\rangle| / \sum_{hkl} \sum_i I_i(hkl)$; [d]root mean square deviation; [e]calculated from the B values of all non-hydrogen atoms; [f]calculated with Molprobity.

Movie S1. Movie of the MD simulation of $sclx_4$ binding to Lys116-Me_2. Each frame corresponds to a 100 ps time point over the 10 ns simulation. The simulation was run in explicit solvent but the waters are not shown. The side chains of Asn106 and Arg112 are shown as sticks. Note the formation of hydrogen bonds and salt bridges between these side chains and $sclx_4$.
